# Feasibility of omitting regional nodal irradiation in cT1–2N1 breast cancer with ypN1 disease after neoadjuvant chemotherapy (KROG 21-06)

**DOI:** 10.1016/j.ctro.2026.101203

**Published:** 2026-05-30

**Authors:** Younghee Park, Kyubo Kim, Kyung Hwan Shin, Ji Hyun Chang, Su Ssan Kim, Jin Hong Jung, Won Park, Haeyoung Kim, Yong Bae Kim, Sung Ja Ahn, Myungsoo Kim, Jin Hee Kim, Hyejung Cha, Tae Gyu Kim, Hae Jin Park, Sun Young Lee

**Affiliations:** a Department of Radiation Oncology, Ewha Womans University College of Medicine, Seoul, the Republic of Korea; b Department of Radiation Oncology, Seoul National University Bundang Hospital, Seongnam, the Republic of Korea; c Department of Radiation Oncology, Seoul National University College of Medicine, Seoul, the Republic of Korea; d Department of Radiation Oncology, Asan Medical Center, University of Ulsan College of Medicine, Seoul, the Republic of Korea; e Department of Radiation Oncology, Samsung Medical Center, Sungkyunkwan University School of Medicine, Seoul, the Republic of Korea; f Department of Radiation Oncology, Yonsei University College of Medicine, Seoul, the Republic of Korea; g Department of Radiation Oncology, Chonnam National University Medical School, Gwangju, the Republic of Korea; h Department of Radiation Oncology, Incheon St. Mary’s Hospital, College of Medicine, The Catholic University of Korea, Seoul, the Republic of Korea; i Department of Radiation Oncology, Keimyung University School of Medicine, Daegu, the Republic of Korea; j Department of Radiation Oncology, Yonsei University Wonju College of Medicine, Wonju, the Republic of Korea; k Department of Radiation Oncology, Samsung Changwon Hospital, Sungkyunkwan University School of Medicine, Changwon, the Republic of Korea; l Department of Radiation Oncology, Hanyang University College of Medicine, Seoul, the Republic of Korea; m Department of Radiation Oncology, Jeonbuk National University School of Medicine, Jeonju, the Republic of Korea

**Keywords:** Breast cancer, Neoadjuvant chemotherapy, Regional nodal irradiation

## Abstract

**Purpose:**

To evaluate the effect of regional nodal irradiation (RNI) in breast cancer patients with 1–3 residual nodal metastases (ypN1) after neoadjuvant chemotherapy (NAC).

**Methods:**

We retrospectively reviewed the medical records of breast cancer patients with clinical T1–2N1 disease who received NAC followed by surgery and postoperative radiotherapy between 2005 and 2017. Patients with 1–3 pathologically positive lymph nodes (ypN1) after axillary dissection were included. Univariate and multivariate analyses were performed to identify the prognostic factors for locoregional recurrence-free survival (LRRFS), distant metastasis-free survival (DMFS), disease-free survival (DFS), and overall survival (OS).

**Results:**

The median follow-up duration was 88.9 months (range, 5.0–200.1). A total of 245 patients were included and RNI was administered in 84.5% (n = 207). Lymphatic invasion and extranodal extension were more frequent in patients who received RNI compared with those who did not (*p* = 0.009 and *p* < 0.001, respectively), while other patient and tumor characteristics were similar between the two groups. On multivariate analysis, histologic grade was identified as a significant prognostic factor for LRRFS (*p* = 0.045) and lymphatic invasion was significantly associated with DMFS (*p* = 0.029). However, RNI was not associated with any of the survival outcomes. The 5-year LRRFS, DMFS, DFS, and OS in the RNI and non-RNI groups were 89.5% vs. 97.2%, 84.2% vs. 91.6%, 81.7% vs. 91.6% and 91.1% vs. 100%, respectively (all *p* > 0.05).

**Conclusion:**

RNI was not associated with additional survival benefit in cT1–2N1 breast cancer patients with ypN1 disease after NAC. These findings warrant further validation.

## Highlights

• The role of RNI was evaluated in cT1-2N1 breast cancer with ypN1 disease after NAC and ALND.
• RNI was not associated with additional survival benefit in this patient group.
• Omission of RNI may be considered in selected patients.

## Introduction

Historically, regional nodal irradiation (RNI) has been indicated for patients with node-positive breast cancer to reduce locoregional recurrence (LRR) and improve survival [1]. Neoadjuvant chemotherapy (NAC) has been widely adopted as a standard of care for locally advanced breast cancer and is increasingly used in earlier-stage disease with nodal involvement. Because NAC could effectively downstage axillary disease, the post-chemotherapy nodal status may no longer reflect the actual risk of recurrence. As a result, the use of NAC has introduced challenges in determining the appropriate indications and extent of adjuvant radiotherapy (RT).

Traditionally, decisions regarding adjuvant RT after NAC have been based on the initial disease status [2]. Earlier evidence evaluating the efficacy of RT in patients treated with NAC and mastectomy demonstrated that postmastectomy RT (PMRT) significantly improved both local control and survival in patients with clinical T3 tumor, stage III-IV disease or four or more positive lymph nodes, suggesting the need of RT regardless of the response to NAC [3].

However, emerging data suggest risk-adapted strategies that incorporate the response to NAC in guiding adjuvant RT decisions. The prospective registry study RAPCHEM investigated the de-escalation of adjuvant RT based on nodal response to NAC in patients with cT1–2N1 breast cancer [4]. Patients were stratified into three risk groups (low, intermediate, and high) based on ypN status and treated according to the predefined recommendations: RNI was omitted in low-risk (ypN0) and in intermediate-risk patients with ypN1 disease after axillary dissection, while high-risk patients with ypN2–3 disease received RNI. The study demonstrated excellent locoregional control across all groups, supporting the safety of tailoring RT according to nodal response after NAC.

Recently, the National Surgical Adjuvant Breast and Bowel Project (NSABP) B-51/Radiation Therapy Oncology Group (RTOG) 1304 trial evaluated the role of RNI in patients with cT1-3N1 breast cancer and biopsy-proven nodal metastasis who converted to ypN0 after NAC [5]. The trial demonstrated that RNI did not improve recurrence-free (RFS) or overall survival (OS) rates, supporting the safety of omitting RNI in this population.

However, data on patients with residual ypN1 disease after NAC remain limited, and the benefit of RNI in these patients has not been clearly defined. Therefore, we aimed to evaluate the impact of RNI on survival outcomes in patients with cT1–2N1 breast cancer who had one to three residual positive nodes following NAC and surgery, corresponding to the intermediate-risk population defined in the RAPCHEM trial.

## Methods

### Patients

We retrospectively reviewed the medical records of breast cancer patients who received NAC followed by surgery between 2005 and 2017 at 12 institutions in Korea. The study protocol was approved by the Institutional Review Board of each participating institution, and the requirement for informed consent was waived.

The inclusion criteria were as follows: (1) cT1-2N1 at diagnosis, (2) pathologically confirmed 1–3 positive axillary lymph nodes (ypN1) after NAC, (3) completion of axillary lymph node dissection (ALND), (4) receipt of postoperative RT to breast or chest wall with or without RNI. Patients with bilateral breast cancer, distant metastasis at diagnosis, a history of other malignancy, or incomplete information regarding the RT field were excluded.

### Statistical analysis

LRR was defined as tumor relapse encompassing local recurrence (ipsilateral breast or chest wall) or regional recurrence (ipsilateral axillary, supraclavicular (SCN), or internal mammary (IMN) lymph nodes). Any recurrence outside these areas was classified as distant metastasis. Locoregional recurrence-free survival (LRRFS), distant metastasis-free survival (DMFS), disease-free survival (DFS), and OS were calculated from the date of surgery to the corresponding event. The differences in categorical variables were compared with the chi-square or Fisher's exact test. Actuarial survival rates were calculated using the Kaplan-Meier method, and differences were compared with the log-rank test. A Cox proportional hazards model was used for multivariate analysis. A *p*-value of <0.05 was considered statistically significant. All statistical analyses were performed using R software (version 4.4.2, https://www.r-project.org/).

## Results

### Patient and tumor characteristics

A total of 245 patients were included in the final analysis. The median age was 47 years (range, 29–72) and 84.5% (n = 207) received RNI.

For NAC, most patients (91.0%, n = 223) received a combination of anthracycline and taxane, while 4.9% (n = 12) received a taxane-based regimen. Breast-conserving surgery was performed in 186 patients and 59 underwent mastectomy. Among those who underwent mastectomy, 47 had total mastectomy, 7 had skin-sparing mastectomy and 5 had nipple-sparing mastectomy. All patients underwent ALND. Of these, 131 patients underwent sentinel lymph node biopsy followed by ALND, while 111 underwent ALND without sentinel lymph node biopsy. The status of sentinel lymph node biopsy was not available in 3 patients.

Of 207 patients who received RNI, 137 received irradiation to the axilla and SCN, whereas 70 received irradiation to the axilla, SCN, and IMN. The prescribed radiation dose ranged from 39 to 50.4 Gy in 13–28 fractions, with a fraction dose of 1.8–3 Gy. Tumor bed boost was delivered in 174 patients, with doses ranging from 9 to 20 Gy in 3–10 fractions at 1.8–3 Gy per fraction.

Patient and tumor characteristics according to the receipt of RNI are summarized in Table 1. All patients in the non-RNI group underwent BCS and all patients who underwent mastectomy received RNI. Lymphatic invasion and extranodal extension were more frequently observed in the RNI group (*p* = 0.004 and *p* < 0.001, respectively). A greater proportion of patients (27.5%) received adjuvant chemotherapy in the RNI group than non-RNI group (10.5%, *p* = 0.043).

**Table 1:** Baseline characteristics according to the regional nodal irradiation.

	total	RNI (−) (N = 38)	RNI (+) (N = 207)	*p-*value
Age, years				0.820
median	47	47	47	
range	29–72	29–72	29–71	
Laterality				0.322
Left	127 (51.8%)	23 (60.5%)	104 (50.2%)	
Right	118 (48.2%)	15 (38.5%)	103 (49.8%)	
Types of surgery				0.000
Breast conservation	186 (75.9%)	38 (100.0%)	148 (71.5%)	
Mastectomy	59 (24.1%)	0 (0.0%)	59 (28.5%)	
cTstage				0.848
cT1	38 (15.5%)	5 (13.2%)	33 (15.9%)	
cT2	207 (84.5%)	33 (86.8%)	174 (84.1%)	
ypTstage				0.367
ypT0	18 (7.3%)	2 (5.3%)	16 (7.7%)	
ypTis	4 (1.6%)	0 (0.0%)	4 (1.9%)	
ypT1	134 (54.7%)	26 (68.4%)	108 (52.2%)	
ypT2	84 (34.3%)	10 (26.3%)	74 (35.7%)	
ypT3	5 (2.0%)	0 (0.0%)	5 (2.4%)	
No of metastatic nodes				0.621
1	118 (57.6%)	21 (55.3%)	97 (46.9%)	
2	79 (32.2%)	11 (28.9%)	68 (32.9%)	
3	25 (19.6%)	6 (15.8%)	42 (20.3%)	
Histologic grade				0.423
High	68 (27.4%)	13 (34.2%)	55 (26.6%)	
Intermediate	137 (55.9%)	22 (57.9%)	115 (55.6%)	
Low	19 (7.8%)	1 (2.6%)	18 (8.7%)	
N/A	21 (8.6)	2 (5.3%)	19 (9.2)	
Resection margin				0.279
Negative	195 (79.6%)	26 (68.4%)	169 (81.6%)	
Close	39 (18.4%)	10 (26.3%)	29 (14.0%)	
Positive	6 (2.4%)	1 (2.6%)	5 (2.4%)	
N/A	5 (2.0%)	1 (2.6%)	4 (1.9%)	
Lymphatic invasion				0.004
Negative	141 (57.6%)	31 (81.6%)	110 (60.1%)	
Positive	79 (32.2%)	6 (15.8%)	73 (39.9%)	
N/A	25 (10.2%)	1 (2.6%)	24 (11.6%)	
Extranodal extension				0.000
Negative	93 (38.0%)	26 (68.4%)	67 (32.4%)	
Positive	46 (18.8%)	7 (18.4%)	39 (18.8%)	
N/A	106 (43.3%)	5 (13.2%)	101 (48.8%)	
Estrogen receptor				0.094
Negative	75 (30.6%)	7 (18.4%)	68 (32.9%)	
Positive	164 (66.9%)	31 (81.6%)	133 (64.3%)	
N/A	6 (2.4%)	0 (0.0%)	6 (2.9%)	
Progesterone receptor				0.367
Negative	102 (41.6%)	14 (36.8%)	88 (42.5%)	
Positive	136 (55.5%)	24 (63.2%)	112 (54.1%)	
N/A	7 (2.9%)	0 (0.0%)	7 (3.4%)	
HER2				0.743
Negative	143 (58.4%)	21 (55.3%)	122 (58.9%)	
Equivocal	25 (10.2%)	5 (13.2%)	20 (9.7%)	
Positive	73 (29.8%)	12 (31.6%)	61 (29.5%)	
N/A	4 (1.6%)	0 (0.0%)	4 (1.9%)	
Subtype				0.250
HR (+)	169 (69.0%)	31 (81.6%)	138 (66.7%)	
HR (−), HER2(+)	27 (11.0%)	3 (7.9%)	23 (11.1%)	
HR (−), HER2(−)	39 (15.9%)	4 (10.5%)	36 (17.4%)	
N/A	10 (4.1%)	0 (0.0%)	10 (4.8%)	
Adjuvant chemotherapy				0.043
No	184 (75.1%)	34 (89.5%)	150 (72.5%)	
Yes	61 (24.9%)	4 (10.5%)	57 (27.5%)	
Adjuvant anti-HER2 therapy				0.190
No	185 (75.5%)	25 (65.8%)	160 (77.3%)	
Yes	60 (24.5%)	13 (34.2%)	47 (22.7%)	
Hormone therapy				0.265
No	78 (31.8%)	8 (21.1%)	70 (33.8%)	
Yes	166 (67.8%)	30 (78.9%)	136 (65.7%)	
N/A	1 (0.4%)	0 (0.0%)	1 (0.5%)	

Abbreviations: RNI = regional nodal irradiation; N/A = not available; HER2 = human epidermal growth factor receptor 2; HR = hormone receptor.

### Patterns of recurrence

The median follow-up duration was 88.9 months (range, 5.0–200.1). There were two cases of local recurrence in the non-RNI group (5.3%) and twelve cases in the RNI group (5.8%). Regional recurrence occurred in two patients (5.3%) who did not receive RNI and in sixteen patients (7.7%) who received RNI. In the non-RNI group, all regional recurrences developed in the axilla. In patients treated with RNI, regional recurrences were located in the axilla alone in 2 patients (1.0%), SCN alone in 3 (1.4%), IMN alone in 2 (1.0%), both axilla and SCN in 5 (2.4%), both IMN and SCN in 2 (1.0%), and in all three regions in 1 (0.5%). Distant metastasis developed in 5 patients (13.2%) in the non-RNI group and in 38 patients (18.4%) in the RNI group. Distant lymph nodes and lung were the most frequent metastatic sites (46.5% each), followed by bone (44.2%) and liver (23.3%).

### Treatment outcomes

The 5-year LRRFS, DMFS, DFS and OS rates were 90.7%, 85.3%, 83.2% and 92.5%, respectively. The results of univariate analysis for LRRFS, DMFS, DFS and OS are summarized in Table 2. Histologic grade was significantly associated with LRRFS (*p* = 0.009), DMFS (*p* = 0.003) and DFS (*p* = 0.003). The presence of lymphatic invasion was significantly associated with inferior DMFS (*p* = 0.008), DFS (*p* = 0.030), and OS (*p* = 0.036). However, no significant association was observed between the receipt of RNI and any of the survival endpoints. On multivariate analysis (Table 3), histologic grade was identified as an independent prognostic factor for LRRFS (*p* = 0.045). In addition, the presence of lymphatic invasion was significantly associated with poorer DMFS (*p* = 0.029).

**Table 2:** Univariate analysis for LRRFS, DMFS, DFS and OS.

	LRRFS			DMFS			DFS			OS	
Variables	5-yr rate	*p*-value		5-yr rate	*p*-value		5-yr rate	*p*-value		5-yr rate	*p*-value

Laterality											
Left	86.0	0.072		82.8	0.298		80.4	0.307		90.3	0.231
Right	95.6			87.9			86.1			94.9	
Breast surgery											
Breast conservation	90.0	0.207		85.1	0.462		82.9	0.378		92.9	0.706
Mastectomy	92.9			86.0			84.3			91.4	
Clinical T stage											
cT1	94.7	0.198		92.1	0.083		92.1	0.040		94.7	0.151
cT2	90.0			84.0			81.5			92.1	
ypT stage											
ypT0-T1	90.2	0.863		86.9	0.282		85.0	0.316		95.4	0.008
ypT2-T3	91.7			82.6			80.1			87.4	
No of metastatic nodes											
1	92.1	0.116		85.3	0.701		84.4	0.479		94.0	0.217
2	91.9			87.0			83.0			93.6	
3	85.1			82.7			80.6			87.2	
Histological grade											
Low/intermediate	93.4	0.009		88.8	0.003		86.8	0.003		94.8	0.121
High	81.3			72.5			69.5			85.0	
Resection margin											
Close/Positive	88.0	0.306		83.7	0.334		78.8	0.367		93.3	0.253
Negative	91.0			85.8			84.3			92.1	
Lymphatic invasion											
Negative	93.4	0.087		91.2	0.008		88.3	0.030		96.4	0.036
Positive	82.9			75.2			73.8			87.1	
Extranodal extension											
Negative	93.4	0.761		89.1	0.449		88.0	0.381		93.5	0.087
Positive	91.0			81.9			81.9			93.3	
Estrogen receptor											
Negative	83.5	0.076		75.3	0.038		72.5	0.040		85.1	0.088
Positive	93.6			89.9			88.0			96.3	
Progesterone receptor											
Negative	86.8	0.466		80.7	0.384		78.7	0.502		87.1	0.174
Positive	93.1			88.7			86.4			96.9	
HER2											
Negative/equivocal	90.2	0.533		85.9	0.488		84.1	0.771		92.1	0.490
Positive	91.4			84.6			81.6			94.4	
Subtype											
HR (+)	93.2	0.062		88.4	0.182		86.6	0.182		95.8	0.110
HR(−), HER2(+)	88.7			81.1			77.2			92.6	
HR(−), HER2(−)	78.4			73.1			70.5			78.8	
Adjuvant chemotherapy											
No	89.4	0.072		84.5	0.121		82.2	0.089		92.8	0.473
Yes	94.8			88.0			86.4			91.7	
Adjuvant anti-HER2 therapy											
No	90.5	0.866		85.6	0.910		83.3	0.938		92.3	0.490
Yes	91.4			84.9			83.1			93.1	
Hormone therapy											
No	85.5	0.233		77.5	0.170		76.2	0.310		85.6	0.180
Yes	93.0			88.8			86.3			95.7	
RNI											
No	97.2	0.407		91.6	0.352		91.6	0.356		100.0	0.170
Yes	89.5			84.2			81.7			91.1	

Abbreviations: LRRFS = locoregional recurrence-free survival; DMFS = distant metastasis-free survival, DFS = disease-free survival; OS = overall survival; HER2 = human epidermal growth factor receptor 2; HR = hormone receptor; RNI = regional nodal irradiation.

**Table 3:** Multivariate analysis for LRRFS, DMFS, DFS and OS.

	LRRFS			DMFS			DFS			OS	
Variables	HR (95%CI)	*p*-value		HR (95%CI)	*p*-value		HR (95%CI)	*p*-value		HR (95%CI)	*p*-value

Laterality											
Left	1.820 (0.806–4.109)	0.149		N/A			N/A			N/A	
Right	1			N/A			N/A			N/A	
Clinical T stage											
cT1	N/A			1			1			N/A	
cT2	N/A			1.480 (0.451–4.856)	0.518		1.835 (0.564–5.972)	0.313		N/A	
ypT stage											
ypT0-T1	N/A			N/A			N/A			1	
ypT2-T3	N/A			N/A			N/A			1.919 (0.728–5.059)	0.188
Histological grade											
Low/intermediate	1			1			1			N/A	
High	2.435 (1.020–5.813)	0.045		1.870 (0.916–3.816)	0.085		1.838 (0.934–3.618)	0.078		N/A	
Lymphatic invasion											
Negative	1			1			1			1	
Positive	1.805 (0.811–4.018)	0.148		2.071 (1.079–3.975)	0.029		1.724 (0.935–3.179)	0.081		1.141 (0.409–3.183)	0.801
Extranodal extension											
Negative	N/A			N/A			N/A			1	
Positive	N/A			N/A			N/A			1.790 (0.668–4.793)	0.247
Estrogen receptor											
Negative	N/A			1.471 (0.712–3.038)	0.298		1.574 (0.788–3.147)	0.199		1.501 (0.524–4.297)	0.449
Positive	N/A			1			1			1	
Subtype											
HR (+)	1			N/A			N/A			N/A	
HR(−), HER2(+)	0.918 (0.249–3.388)	0.898		N/A			N/A			N/A	
HR(−), HER2(−)	2.213 (0.824–5.946)	0.115		N/A			N/A			N/A	
Adjuvant chemotherapy											
No	2.508 (0.721–8.721)	0.148		N/A			2.199 (0.847–5.715)	0.106		N/A	
Yes	1			N/A			1			N/A	
RNI											
No	1			1			1			1	
Yes	1.787 (0.505–6.328)	0.368		1.371 (0.517–3.633)	0.526		1.478 (0.601–3.633)	0.395		1.846 (0.501–6.806)	0.357

Abbreviations: LRRFS = locoregional recurrence-free survival; DMFS = distant metastasis-free survival, DFS = disease-free survival; OS = overall survival; HER2 = human epidermal growth factor receptor 2; HR = hormone receptor; RNI = regional nodal irradiation, N/A = not assessed.

**Fig. 1:**
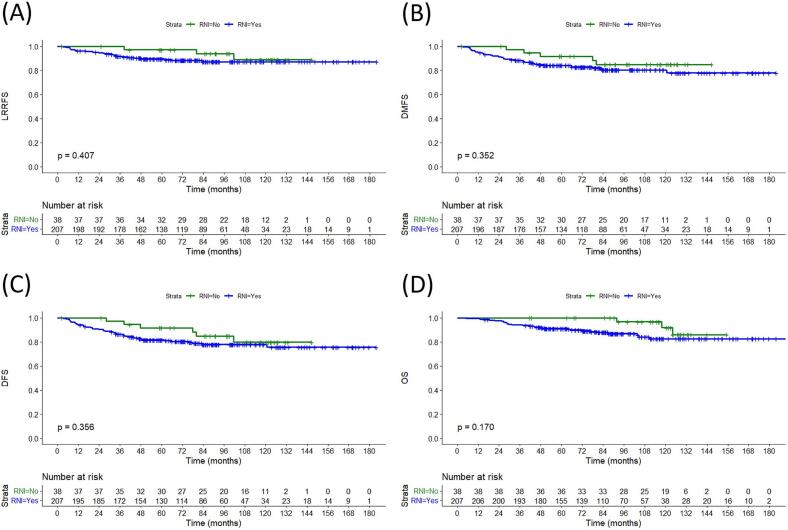
Kaplan-Meier survival curves for locoregional recurrence-free survival (A), distant metastasis-free survival (B), disease-free survival (C), and overall survival (D).

The 5-year LRRFS, DMFS, DFS, and OS rates were similar between the RNI and non-RNI groups (89.5% vs. 97.2% for LRRFS, 84.2% vs. 91.6% for DMFS, 81.7% vs. 91.6% for DFS and 91.1% vs. 100% for OS; all *p* > 0.05; Fig. 1).

## Discussion

In this multicenter retrospective study involving patients with cT1–2N1 breast cancer who had one to three residual axillary metastases after NAC, RNI did not improve LRRFS, DMFS, DFS, or OS.

Decisions regarding RT indications and fields after NAC have been largely extrapolated from outcomes of upfront surgery. Meanwhile, given the response to NAC varies substantially among patients and is strongly associated with prognosis, there is a growing need for tailoring RT according to treatment response. However, due to the limited evidence, standardized guidelines have not yet been established, resulting in considerable variability in clinical practice [6], [7].

A retrospective study reported the impact of PMRT in patients with stage II–III breast cancer treated with NAC and mastectomy [8]. In this study, PMRT was administered in 87.4% of the included patients, and among those who underwent PMRT, 97.0% received RNI. In patients with ypN0 after NAC, PMRT did not improve the overall treatment outcomes but was associated with improved LRRFS in the subgroup with the triple-negative subtype. Among patients with residual nodal disease, PMRT significantly improved LRRFS, DFS, and OS. However, within the ypN1 subgroup, the benefit of PMRT was observed in patients with two or more risk factors, including a molecular subtype other than luminal/HER2-negative, histologic grade III, and the presence of lymphovascular invasion.

Additionally, several retrospective studies have evaluated the role of PMRT and RNI after NAC. Although their findings have been somewhat inconsistent, many have demonstrated the safety of omitting PMRT or RNI in patients who achieve ypN0 status [9], [10], [11]. Recently, the results of prospective randomized NSABP B-51/RTOG 1304 trial have been reported [5]. In this study, patients with biopsy-proven, node-positive breast cancer who achieved ypN0 status after NAC were randomly assigned to receive RNI or no RNI. After a median follow-up of 59.5 months, RNI did not improve RFS or OS, demonstrating the safety of RNI omission in this population.

In contrast, while growing evidence supports RT de-escalation in patients who achieve ypN0 status, the optimal extent of adjuvant RT in patients with ypN1 remains uncertain. The ACOSOG Z1071 trial showed that LRR decreased with PMRT or the addition of RNI to whole-breast RT in patients with residual nodal disease (ypN+). However, patients with ypN1 disease were not analyzed separately [12]. A population-based study found no OS benefit of RNI in patients with minimal residual nodal disease, such as isolated tumor cells or micrometastases after NAC [13]. A recent SEER-based analysis suggested that PMRT should be recommended for patients with 3 residual node metastases, while in those undergoing breast-conserving surgery or mastectomy with 1–2 positive nodes, sentinel lymph node biopsy combined with RT could replace ALND [14]. Therefore, additional risk stratification may be necessary for ypN1 patients who do not undergo ALND.

The RAPCHEM study proposed a risk-adapted RT strategy that incorporates nodal response after NAC, axillary surgical management, and predefined risk factors [4]. The intermediate-risk group consisted of patients with ypN1 disease who underwent ALND, as well as patients managed without ALND who met predefined nodal and risk factor criteria. RT recommendations differed according to axillary management: ypN1 patients treated with ALND were recommended to receive whole-breast or chest wall RT alone, while intermediate-risk patients who did not undergo ALND were recommended to receive RT including axillary levels I and II.

Notably, in the RAPCHEM study, adherence to the study guideline in the intermediate-risk group was 54%. In a post-hoc analysis accounting for guideline adherence, RT intensification did not provide additional benefit; the 5-year LRR and OS rates were 1.0% and 95.0% among patients treated according to the guideline and 3.8% and 91.4% among those who received more RT than recommended. These findings suggest the oncologic safety of RNI omission in intermediate-risk patients.

The present study included only ypN1 patients who underwent ALND, thereby constituting a clinically homogeneous cohort corresponding to the intermediate-risk subgroup defined in the RAPCHEM study. Consistent with the RAPCHEM result, no significant differences in treatment outcomes were observed according to the receipt of RNI, with 5-year LRRFS rates of 89.5% and 97.2% and 5-year OS rates of 91.1% and 100% in the RNI and non-RNI groups, respectively. Although the locoregional control appeared slightly lower in the present study, it should be noted that the RAPCHEM study excluded LRR with synchronous DM from the LRR analysis. When such cases were included, the reported LRR rates in the RAPCHEM study ranged from 4.9% to 5.8%, which are comparable to those observed in the present study. Overall, RNI did not improved the treatment outcomes, thereby supporting the de-escalation of RT in this patient population, as proposed by the RAPCHEM strategy. A comparison between the RAPCHEM study and the present study is summarized in Supplementary Table 1.

This study has several limitations inherent to its retrospective design. The administration of RNI was not randomized, and physicians may have determined the treatment volume based on perceived patient risk, which may have resulted in selection bias. Therefore, the majority of patients (84.5%) received RNI, and omission of RNI may have been influenced by favorable prognostic features that were not accounted for in the present analysis. Consistent with this, although the differences were not statistically significant, patients who received RNI tended to have worse DMFS, DFS, and OS compared with those who did not. Furthermore, the number of patients in the non-RNI group was relatively small (n = 38), and the limited number of events in this group may have reduced the statistical power to detect potential differences in outcomes.

Some pathologic factors, such as histologic grade, resection margin status, and lymphatic invasion, were not available in some patients. Because a substantial portion of the tumor regresses after NAC, assessment of these pathologic factors may have been challenging. Additionally, only a small number of patients belonged to the hormone receptor (HR)–negative/human epidermal growth factor receptor 2 (HER2)-positive and HR-negative/HER2-negative subtypes (n = 27 and n = 39, respectively), and since most of these patients received RNI, differences in prognosis according to subtype could not be evaluated.

Moreover, the study period extended from 2005 to 2017. During this interval, significant changes occurred in systemic treatment, surgical techniques, RT practice, and pathologic evaluation standards. These changes in treatment approaches over time may have influenced treatment decisions and outcomes, necessitating cautious interpretation of the results. In addition, although the median follow-up duration was 88.9 months (range, 5.0–200.1), this may be insufficient to fully capture late recurrences in HR-positive breast cancer, which are known to occur beyond 10 years.

Despite these limitations, our study included a homogeneous cohort of patients with initial cT1–2N1 disease who all underwent axillary dissection and were found to have residual metastases in 1–3 axillary lymph nodes, with a relatively long follow-up period. In this intermediate-risk group, RNI was not associated with improved treatment outcomes, which is in line with the findings of the RAPCHEM study. Our results may be interpreted as a hypothesis-generating contribution to future studies evaluating risk-adapted RT de-escalation strategies in intermediate-risk patients after NAC. Future well-designed prospective studies involving a larger number of patients are warranted to validate these findings.

## CRediT authorship contribution statement

**Younghee Park:** Data curation, Formal analysis, Visualization, Writing – original draft, Writing – review & editing. **Kyubo Kim:** Conceptualization, Investigation, Methodology, Supervision, Writing – review & editing. **Kyung Hwan Shin:** Conceptualization, Investigation, Methodology, Supervision, Writing – review & editing. **Ji Hyun Chang:** Data curation, Resources. **Su Ssan Kim:** Investigation, Resources. **Jin Hong Jung:** Data curation, Resources. **Won Park:** Investigation, Resources. **Haeyoung Kim:** Data curation, Resources. **Yong Bae Kim:** Investigation, Resources. **Sung Ja Ahn:** Investigation, Data curation, Resources. **Myungsoo Kim:** Investigation, Data curation, Resources. **Jin Hee Kim:** Investigation, Data curation, Resources. **Hyejung Cha:** Investigation, Data curation, Resources. **Tae Gyu Kim:** Investigation, Data curation, Resources. **Hae Jin Park:** Investigation, Data curation, Resources. **Sun Young Lee:** Investigation, Data curation, Resources.

## Funding

This work was supported by the National R&D Program for Cancer Control through the National Cancer Center (NCC) funded by the Ministry of Health & Welfare, Republic of Korea (HA22C0044).

## Declaration of competing interest

The authors declare that they have no known competing financial interests or personal relationships that could have appeared to influence the work reported in this paper.
