# Supplementary material for: Feasibility of omitting regional nodal irradiation in cT1–2N1 breast cancer with ypN1 disease after neoadjuvant chemotherapy (KROG 21-06)
Source: Clin Transl Radiat Oncol. 2026 May 30;60:101203. doi: 10.1016/j.ctro.2026.101203 (PMC13266224; doi:10.1016/j.ctro.2026.101203)
Supplement: Supplementary Data 1 [file mmc1.docx]

Supplementary Table 1. Comparison between the RAPCHEM study and the present study

|  | RAPCHEM study (intermediate-risk group) | Present study |
| --- | --- | --- |
| Study design | Prospective multicenter registry | Retrospective multicenter cohort |
| Study population | cT1–2N1 breast cancer treated with NAC | cT1–2N1 breast cancer treated with NAC |
| Initial nodal status | At least one pathologically confirmed metastatic lymph node | Not restricted |
| Axillary surgery | ALND or SLNB/MARI | ALND |
| RT strategy | ypN1 and ALND  : whole-breast or chest wall RT only    no ALND &  if SLNB before NAC  cN1mi, ≥1 risk factor*  cN1, ≤2 macrometastases, no risk factor  if SLNB after NAC  ypN1mi, no risk factor  : whole-breast or chest wall RT + axilla level I and II | Breast or chest wall RT ± RNI |
| Guideline adherence / RNI use | Guideline adherence : 54.0%  Less RT than guideline : 17.0%  More RT than guideline : 29.0% | No RNI : 15.5%  RNI : 84.5 % |
| Locoregional outcome & OS | 5-year LRR & OS rates  Overall patients : 2.2% & 94.0%  According to guideline : 1.0% & 95.0%  More RT than guideline 3.8% & 91.4% | 5-year LRRFS & OS rates  No RNI : 97.2% & 100%  RNI : 89.5% & 91.1% |

*risk factor : grade 3, lymphovascular invasion, tumor size more than 3cm

Abbreviations : NAC = neoadjuvant chemotherapy; ALND = axillary lymph node dissection; SLNB= sentinel lymph node biopsy; MARI = marking the axillary lymph node with radioactive iodine seed; RT=radiotherapy; LRR=locoregional recurrence; LRRFS=locoregional recurrence-free survival; RNI= regional nodal irradiation.
